# Teachers’ and Parents’ Assessment of Challenges in Children Exhibiting Sensory Seeking Behavior and Possible Effects of the Use of Ball Vests: A Pre–Post Study

**DOI:** 10.3390/children10111800

**Published:** 2023-11-10

**Authors:** Ann Natasja Nielsen, Karen la Cour, Åse Brandt

**Affiliations:** 1 National Institute of Public Health, The Faculty of Health Sciences, University of Southern Denmark, 1455 Copenhagen, Denmark; 2 Occupational Science, Research Unit for User Perspective and Community-Based Intervention, Department of Public Health, University of Southern Denmark, 5000 Odense, Denmark

**Keywords:** sensory integration, sensory processing, school participation, primary school, assistive technology

## Abstract

Children with dysfunction in sensory processing (DSP) may experience challenges, which might affect their participation in activities and potentially also their further development. This study examined the challenges of children with DSP who exhibit sensory seeking behavior, the differences in these challenges between boys and girls, and the possible effects of their use of ball vests. The challenges of 70 pupils (aged 6–12 years) exhibiting sensory seeking behavior were assessed by their parents (n = 66) and teachers (n = 60) by surveys containing items from the ‘Strengths and Difficulties Questionnaire’ (SDQ) and the ‘Five to Fifteen’ (FTF) questionnaire. Differences in the SDQ/FTF scores between boys and girls were explored using chi-square analysis. The potential effects of the ball vest were assessed using a study-specific follow-up survey. Linear mixed model regression analysis was used to examine associations between the extent of use of the vest and the assessed effects. The pupils were assessed for challenges that interfered with their learning (62%), forming friendships (51.7%), and the classroom environment (56.9%). After three weeks, the parents found that some pupils had improved regarding attention (39%), body perception (34%), and hyperactivity and impulsivity (33%). The teachers reported that 30% of the pupils had improved their coping skills in learning.

## 1. Introduction

Sensory processing comprises the neurological processes by which people organize, regulate, and respond to sensory information [1,2]. Studies suggest that dysfunctions in sensory processing (DSP) affect children’s choice of—and participation in—everyday activities and school activities [3,4,5]. Furthermore, children with DSP were to have decreased levels of enjoyment from participating in everyday activities [6]. A review of performance difficulties (i.e., in school, leisure, or everyday activities) for children with DSP found that these children had a variety of performance difficulties that differed between children with different subtypes of DSP [7]. Researchers recommend that further research should focus on performance difficulties for specific subtypes of DSP [7,8] and that studies regarding children with DSP should include children without diagnoses related to DSP [9].

The prevalence of DSP among children ranges from 5% to 20% for preschool and primary school children [10,11,12,13]. In India, the prevalence of DSP among children with difficulties in learning was found to be <50%, with about 80% of the children having difficulties in the subtype of sensory seeking behavior [14]. A Danish cross-sectional study showed that about 20% of primary school children had DSP and that 70% of these had difficulties in the subtype of sensory seeking behavior [12]. Therefore, the present study focused on children with DSP and difficulties in the subtype of sensory seeking behavior (referred to as children exhibiting sensory seeking behavior). Research regarding the challenges of children exhibiting sensory seeking behavior is scarce, but these children are known to seek out frequent movement [2], which may affect their ability to participate in learning activities in school and in their everyday lives. Sex is a known determinant of participation in activities among children with DSP, since girls with DSP and boys with DSP have been found to engage in different activities [4,5]. Hence, a focus on the differences in possible challenges among girls and boys can be regarded as relevant.

School-based therapists commonly use deep touch pressure therapy in order to support children with DSP [15,16]. One way to provide deep touch pressure while the children participate in school activities is with garments such as ball vests. One such ball vest was used for the purpose of this study, the Protac MyFit^®^ ball vest (referred to as the vest) (Protac, Skanderborg, Denmark), classified as clothing for sensory stimulation in the International Classification of Assistive Technology ISO 9999:2022 [17]. These vests are composed of weighted plastic balls (either 25 mm or 38 mm in diameter) that are sewn into vertical compartments at the front and back of the vest. The vest weighs between 1 and 2.5 kg depending on its size [18]. When wearing the vest, the deep touch pressure from the balls provides the child with proprioceptive and tactile stimuli, which may reduce functional challenges for children exhibiting sensory seeking behavior, regardless of whether they have a diagnosis.

Sufficient proprioceptive and tactile stimuli are hypothesized to provide children with better body awareness and a sense of calm and collectedness [1,15,19]. Research suggests that such feelings of calm and collectedness can have a positive effect on the ability to pay attention in children with DSP [11,15,16,19] and on off-task behavior [15]. Studies of deep touch pressure with weighted vests in children with attention deficit hyperactivity disorder (ADHD) or autism found these vests to have small to medium effects on children’s attention [15,19].

It is recommended to include the perspectives of the parents (and other relevant stakeholders) when researching how challenges or an intervention may possibly influence a child and its environment [20], for instance by using an instrument such as the Strengths and Difficulties Questionnaire (SDQ) that includes the perspectives of parents and teachers [21]. Experience from practice showed positive effects of using the vest, [22,23], but research on potential effects for children exhibiting sensory seeking behavior without a related diagnosis has, to our knowledge, not been conducted.

The aims of this study were to examine the challenges of children exhibiting sensory seeking behavior, the possible differences in these challenges between boys and girls, and whether use of the vest could reduce these challenges, as assessed by the children’s parents and teachers.

## 2. Materials and Methods

This study is part of a project registered at Clinicaltrials.gov registration number: NCT04173871, first registration date: 21 November 2019. The feasibility of an intervention with use of the vest was tested and evaluated [24]. Permission for the study was sought from the National Committee on Health Research Ethics, and the study was deemed not to be subject for notification to the scientific ethics committee system, according to §14 cf. 1 of the Act on Science Ethical Treatment of Health Science Research Projects case no. S-20182000-155. Permission to obtain and store data was sought from and approved by the Danish Data Protection Agency journal no. 10.135. The study was conducted in accordance with the ethical principles and recommendations from the Declaration of Helsinki 2008 [25]. Additionally, this study adheres to the SAGER guidelines for sex and gender equity in research, with sex defined as the anatomical sex (i.e., binary—boys and girls) [26]. Consent for the children was provided by their parents; additionally, children, teachers, and parents were informed that there were no consequences if the children chose to stop using the vest.

### 2.1. Participants

The participants were the teachers and parents of pupils who participated in a pragmatic randomized controlled study (PRCT) [27] and were allocated to using the vest.

Criteria for inclusion: parent or teacher of pupils aged 6 to 12 years exhibiting sensory seeking behavior, i.e., with a total Short Sensory Profile (SSP) score of 38–154 and SSP section scores regarding sensory seeking behavior of 7–26, attending public school and allocated to using the vest in the PRCT [27].

Criteria for exclusion: parent or teacher of pupils with intellectual and developmental disability or other cognitive impairment attending special needs classes or with severe sensory disability (i.e., blindness or deafness), since these children would not be able to complete the outcome measurements used in the PRCT.

The pupils were recruited from 25 schools in eight Danish municipalities. The participating schools varied in size, with 75 to 1000 pupils per school, and in location (i.e., both rural schools and urban schools).

### 2.2. Study Procedures

As part of the introduction to the study, the teachers at the participating schools received a 90 min lecture regarding DSP, the use of the vest, and how to support pupils’ attention in the classroom. The lectures were provided by the first author (i.e., ANN, Cand. San. in Occupational Therapy, experienced in working with sensory processing in school settings) and held at the different schools (except for three lectures that were held online due to gathering restrictions during the COVID-19 pandemic). Attendance to the lecture was mandatory for teachers at the primary school level, to ensure a common knowledge between the teachers across subject-specific classes and for the recruitment of relevant pupils. Afterwards, the teachers were asked to consider pupils who might exhibit sensory seeking behavior for inclusion in the study and to hand out introduction materials to the parents of the relevant pupils. The introduction materials included a written introduction about the study, a demographic questionnaire, the SSP questionnaire, and a consent form.

The SSP was used to examine whether the pupils exhibited sensory seeking behavior. The SSP is a short version of the 125-item Sensory Profile questionnaire, created for screening programs and research, containing 38 items that were selected from the Sensory Profile questionnaire as the most indicative of sensory processing issues affecting performance [28]. A study showed the discriminant validity of the SSP compared to the Sensory Profile questionnaire to be >95% in identifying children with and without DSP [29]. This study used the Danish translation of the SSP. The parents assess on a five-point Likert scale 38 statements regarding how often they observe the described behavior in their child, resulting in a score from 38 to 190 [28]. SSP scores < 155 indicate DSP. The SSP has a section where seven items indicate whether the child presents sensory seeking behavior; section scores < 26 indicate sensory seeking behavior.

The parents filled out the questionnaires and a consent form, and all eligible pupils were enrolled in a PRCT where they were randomized either to a group using the vest or to a control group. ’The participants in this study were the parents and teachers of pupils allocated to using the vest. The pupils were introduced to the vest by a research assistant and chose whether they wished to use a vest with balls that were 25 mm or 38 mm in diameter, depending on which they found most comfortable. Based on findings from a feasibility study utilizing the vest [24], the pupils were requested to use the vest for the first 90 min of every school day. Besides this, the pupils were invited to use the vest as much as they liked, both at home and at school. Based on findings from a Swedish pilot study [22], the follow-up was determined to be conducted after three weeks.

To examine the challenges of the pupils exhibiting sensory seeking behavior, the parents and the teachers of the enrolled pupils answered an online survey at baseline that contained items of the standardized questionnaire Five to Fifteen (FTF). Additionally, the survey for the teachers’ included items from the Strengths and Difficulties Questionnaire (SDQ). After three weeks, the parents and the teachers answered an online follow-up survey regarding the use of the vest and potential changes (i.e., whether there had been an increase, no change, or a decrease) in the challenges reported at baseline.

### 2.3. Measurements

The FTF questionnaire is a standardized questionnaire for parents and teachers that provides an overview of a child’s neuropsychological and behavioral functions and examines the child’s development in different areas. The FTF questionnaire was developed by experienced practitioners and researchers from Sweden, Denmark, Norway, and Finland. It contains 181 items divided into the following primary domains: motor skills, executive functions (inclusive attention), perception, memory, language, learning, social skills, and emotional/behavioral problems. Each primary domain contains subdomains with 3–13 items (in all 20 subdomains) [30]. The FTF questionnaire showed internal consistency in both domains and subdomains, with Cronbach’s alpha ranging from 0.78 to 0.96 regarding the domains and from 0.69 to 0.94 regarding the subdomains, which indicates a sufficiently high internal consistency [31]. Each item is assessed as ‘does not apply’ (0), ‘applies sometimes or to some extent’ (1), or ‘definitely applies’ (2). The results of the FTF questionnaire are assessed and compared according to a norm sample based on the results from 1350 children [31]. Parents and teachers are asked to assess whether difficulties in each subdomain affect the child’s functions in their everyday life on a scale with the answers ‘not at all’ (0), ‘a little’ (1), ‘some’ (2), and ‘a lot’ (3) [31]. In this study, we included the domain regarding executive functions (subdomains of attention, hyperactive/impulsive, hypoactive, planning/organizing) and the subdomain of body perception in the survey for the parents (in all, 35 items). Additionally, we included the domain regarding learning (subdomains—reading/writing/math, general learning, coping in learning) in the survey for the teachers (in all, 32 items).

The SDQ is a standardized questionnaire for teachers and parents that screens for positive and negative psychological attributes in children aged 3–16 years. The questionnaire consists of 25 items regarding emotional symptoms, conduct problems, hyperactivity–inattention, and social behavior. A review examining the psychometric properties of the SDQ found it to be a strong instrument, especially the teacher version [32]. A study examining the factor structure of the Danish version of the SDQ found that the model fits were slightly better for teachers than for parents and better for older children than for younger children [33]. In this study, we used the 13 items of the SDQ regarding school activities and social behavior for the teachers. Additionally, the teachers assessed whether the collected challenges reported on the SDQ interfered with the pupil’s everyday life (distressed the pupil, affected friendships, affected learning, or affected the classroom environment) on a scale with the answers ‘not at all’, ‘only a little’, ‘quite a lot’, and ‘a great deal’ [21].

To examine changes and the potential effects of the vest, the parents and the teachers were asked in a follow-up survey to assess whether they had observed a change in each of the predefined subdomains of the FTF questionnaire that they had reported as being challenging for the pupil before the use of the vest, evaluating it on a five-point scale as much worse (−2), a little worse (−1), the same (0), a little better (1), and much better (2).

This follow-up survey also included study-specific questions about the extent of use of the vest—i.e., whether the pupil used the vest for the requested 90 min every school day (from the teacher survey) and whether the pupil used the vest additionally at school (from the teacher survey) and/or at home (from the parent survey).

### 2.4. Analysis

Descriptive statistics (mean, standard deviation, count, and percentage) were used to summarize the participants’ characteristics and challenges reported on the SDQ and the FTF questionnaire, using the statistical software IBM SPSS statistics version 28 [34]. Further data analysis was conducted by means of Stata 17.0 [35]. Differences between girls and boys in the scores of all items of the SDQ and of subdomains of the FTF questionnaire were examined using chi-square analysis; when the analysis had an expected count of less than five, the Fisher–Freeman–Halton exact test was used to limit possible type I errors [36]. Missing data were discarded in the analysis. The data from the follow-up survey with the FTF questionnaire were summarized to assess the possible effects of the vest. To examine the association between the extent of use of the vest and its possible effects on the FTF questionnaire, linear regression analysis was used [37]. The dependent variable was the assessed progress on the FTF questionnaire at follow-up, and the independent variable was the use of the vest as reported in the follow-up survey, with the reference being zero-type use. The extent of use of the vest was classified in four categories: no reported use (zero type), 90 min at the beginning of every school day (one type), 90 min at the beginning of every school day and additionally at school or at home (two types), and 90 min at the beginning of every school day and additionally at school and at home (three types).

The statistical significance level was set at *p* < 0.05. To adjust for possible multiple significance, the Bonferroni correction was applied to the analyses of the differences between boys and girls on the SDQ and FTF data [37]. The Bonferroni correction led to reduced significance levels obtained by dividing the statistical significance level of 0.05 by the number of analyses made for each instrument used (i.e., 0.05/13 for SDQ data and 0.05/16 for FTF data).

## 3. Results

The participants were 62 parents and 49 teachers of 70 pupils exhibiting sensory seeking behavior. Table 1 presents the demographic characteristics, diagnostic characteristics, and SSP scores of the pupils.

**Figure 1:**
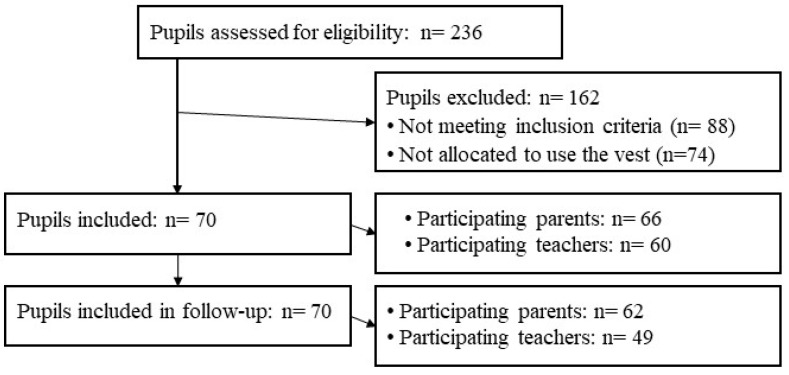
Flowchart showing the selection of the participants.

A flowchart showing the selection of the participants and pupils is presented in Figure 1.

The possible challenges of the pupils related to school activities and social behavior on the SDQ are presented in Table 2. The teachers reported that 78.3% of the pupils were easily distracted and/or let their concentration wander, and 71.7% of the pupils were constantly fidgeting or squirming. Additionally, 66.7% of the pupils were assessed as restless, overactive, and not able to stay still for long. Significant differences were found between boys and girls in challenges regarding restless/overactive (*p* = 0.004) and fidgeting behavior (*p* < 0.001), with boys being assessed as showing these behaviors more often than girls. Furthermore, there was a significant difference between boys and girls regarding their ability to see tasks through to the end (*p* < 0.001), with girls being assessed to have this behavior more often than boys (Table 2).

Table 3 presents the teachers’ assessments of whether the challenges reported on the SDQ interfered with the pupils’ everyday life. They reported that the challenges interfered with learning in 62% of the pupils and with forming friendships in 51.7% of them. Furthermore, for 56.9% of the pupils, the challenges were assessed to interfere with the classroom environment, distracting classmates and/or teachers. A significant difference was found between boys and girls regarding the extent to which the challenges affected the pupils’ learning (*p* = 0.007) and the classroom environment (*p* = 0.026), with the boys being more affected than the girls.

The FTF questionnaire results showed that the parents and the teachers assessed that the pupils had challenges concerning attention, hyperactivity–impulsivity, planning, and coping skills in learning (Table 4). The challenges regarding attention, hyperactivity–impulsivity, planning, and coping skills in learning were especially perceived to affect the pupils’ everyday life. Significant differences between boys and girls were found regarding the challenges of coping skills in learning (*p* = 0.002), with boys being more affected than girls (Table 4).

The results of the follow-up survey showed that 39% of the parents reported that the pupils were able to pay increased attention after being provided with the vest, 34% reported that the pupils had improved body perception, and 33% reported that the pupils sowed improved behavior with respect to hyperactivity and impulsivity. Similarly, the teachers reported that 30% of the pupils showed improved coping skills in learning after being provided with the vest.

No significant difference was found between boys and girls regarding the possible effects of using the vest (Table 5).

In total, 40 of the 70 pupils used the vest for the requested 90 min at the beginning of every school day throughout the three-week period, 26 further used it during school, and 38 used the vest at home. The results showed that the pupils who used the vest for more time than the requested 90 min in every school day were reported to achieve greater assessed progress on the FTF questionnaire (Table 6). The pupils who used the vest for 90 min and additionally either at school or at home showed greater assessed progress than the pupils who did not use the vest for 90 min (B = 1.81, *p* = 0.023). Furthermore, the pupils who used the vest for the requested 90 min and additionally at school and at home showed an even higher assessed progress on the FTF questionnaire (B = 2.48, *p* = 0.007) (Table 6).

## 4. Discussion

The aim of this study was to examine the challenges of pupils exhibiting sensory seeking behavior, the possible differences in the challenges between boys and girls, and whether the use of the ball vest could reduce these challenges, as assessed by the pupils’ parents and teachers. The results showed that the pupils were subjected to a variety of challenges, with some difference between boys and girls, and that the vest could reduce some of these challenges for some of the pupils.

More specifically, the results showed that that the pupils faced challenges particularly regarding attention and concentration, suggesting that the development of interventions for children exhibiting sensory seeking behavior should focus on these challenges. Additionally, the pupils were reported to face challenges regarding hyperactive and fidgeting behavior, which are types of known frequent movement in children exhibiting sensory seeking behavior [1,2]. The results also showed that the pupils scored higher on the FTF questionnaire (i.e., were more challenged) than non-referred children with inattention and hyperactivity–impulsivity included in another Danish study in 2010 [38], indicating that children exhibiting sensory seeking behavior might need more support to participate in school activities than children with other behavioral profiles.

The results of this study showed that the pupils reported a variety of challenges affecting their everyday life and important developmental activities at school, regarding their learning (62.7%), their forming friendships (51.7%), and their interfering with the classroom environment, distracting classmates and/or teachers (56.9%). Pupils’ ability to learn while at the primary school level forms the basis for their further education [39]; challenges in learning can thus potentially influence their future opportunities in life. Pupils can face challenges affecting their own opportunities in life, but by interfering with the classroom environment, they also affect the opportunities of their classmates. The results indicated that boys exhibiting sensory seeking behavior faced greater challenges affecting their learning than girls exhibiting sensory seeking behavior (*p* = 0.007), which might be due to boys with emotional or behavioral problems being behind their peers in academics [39]. This finding suggests that efforts to support boys with sensory seeking behavior could, in particular, have an impact on their learning and academic skills.

The results regarding the possible effects of using the vest as assessed by teachers and parents showed that some pupils reported improved attention (39%), improved body perception (34%), and improved coping skills in learning (30%) after being provided with the vest for three weeks, while no worsening of these functions was reported. This could indicate that the vest may be a tool to reduce some of the challenges of children exhibiting sensory seeking behavior, though further research into the characteristics of children who might benefit from using the vest is needed. The results of the possible effects of using the vest obtained with tests of pupils’ attention, occupational competence, and off-task behavior are reported elsewhere [27].

Of the 70 pupils, 27 did not use the vest as requested, and the results showed no significant effect for the pupils who had used the vest for only the requested 90 min a day. Among the pupils who used the vest for additional time, however, the greatest assessed progress was found for those who used the vest the most. Further research is needed to examine whether the results regarding the assessed progress were limited because 90 min a day was too short a time, three weeks of use was too short a period, or the pupils benefitting from the vest chose to use it more than the pupils who did not benefit from it. The latter could indicate that children exhibiting sensory seeking behavior might benefit from pediatric therapists providing them with an opportunity to use the vest and evaluating its potential effects in close collaboration with the child and possibly their teacher and/or parents.

### Methodological Considerations

When interpreting the results of this study, some limitations should be considered. For instance, the lack of a control group allocated based on random assignment might abate the validity of results regarding the potential effects of the vest. The analysis of the potential effects of the vest was conducted based on self-reported data regarding the extent of the vest use, which was not precise but reported as yes/no answers in three defined categories: ‘90 min daily use in school’, ‘additional use in school’, and ‘additional use at home’. Hence, the pupils reported as not using the vest could potentially have used the vest for up to 89 min a day or as requested for two rather than three weeks. This may have diluted the analysis of the possible effects of using the vest. Furthermore, the sample of 70 pupils may be regarded as relatively small, and a selection bias could have occurred [37], specifically because the teachers might have offered participation to the most disturbing, rowdy, or misbehaving children, while quieter children exhibiting sensory seeking behavior may have been overlooked.

This study had a small representation of girls, which is why the credibility of the statistical analysis of the differences between boys and girls is limited [37]. Lastly, for clarity, the ordinal-scale results of the FTF questionnaire were reported as numerical values with mean and standard deviation, which might provide less nuanced results but adheres to former reporting of FTF results [39].

The results are believed to be representative for Danish pupils and pupils in similar settings in other countries exhibiting sensory seeking behavior, due to the diversity in the demographic characteristics of the participants and the schools they attended. Missing data were discarded but, due to the high response rate (85.71% of teachers and 94.29% of parents), the results regarding the challenges of the pupils, are considered to be credible. Additionally, given the comprehensive character of the surveys and the use of validated materials (i.e., the FTF questionnaire and the SDQ), combined with the duality of the evaluation perspective (i.e., teachers and parents), the results are regarded as adequate. To our knowledge, this is the first Scandinavian study focusing on the challenges of children exhibiting sensory seeking behavior and the assistive technology that may reduce them.

## 5. Conclusions

The results of this study indicate that children exhibiting sensory seeking behavior experience a variety of challenges affecting their everyday life regarding participating in school activities, learning, and forming friendships. The study showed that these children had problems especially with attention and that their sensory seeking behavior interfered with the classroom environment, indicating that interventions to reduce their sensory seeking behavior can have beneficial effects for both the affected child and the general classroom environment. The results suggest that the use of the vest may have a positive effect on some children’s ability to pay attention and that a 90 min daily use of the vest may be too short a time, since among the pupils using the vest, those who used it for longer reported greater improvements. Further research on children exhibiting sensory seeking behavior should include randomly allocated control groups and seek to examine the characteristics of children who might benefit from assistive technology.

## Figures and Tables

**Table 1:** Demographic and diagnostic characteristics of the pupils (N = 70).

	Boys (n = 51)	Girls (n = 19)	Total Sample (N = 70)
Sex	73.2%	26.8%	
Mean age, years (SD)	9.28 (1.82)	8.50 (1.56)	8.93 (1.70)
SSP score, Mean (SD)	132 (17.05)	130 (16.04)	130 (16.01)
Diagnosis % (n) *			
None	73.1 (38)	68.4 (13)	71.8 (51)
ADHD	17.3 (9)	15.8 (3)	17.1 (12)
Autism	5.8 (3)	0.0 (0)	4.3 (3)
Born premature	3.9 (2)	0.0 (0)	2.9 (2)
Other **	9.6 (5)	15.8 (3)	11.4 (8)

SSP = Short Sensory Profile scores (ranging from 38–190), with scores of 142–155 indicating probable challenges, and scores <142 indicating definite challenges with processing sensory information. ADHD = attention deficit hyperactivity disorder. * Some pupils had more than one diagnosis. ** Epilepsy, tremors, arthritis, attention deficit disorder, Tourette’s syndrome, asthma and learning difficulties.

**Table 2:** Teachers’ assessments regarding school activities and social behavior on the Strengths and Difficulties Questionnaire and differences between boys and girls (N = 70).

	Total (n = 60 *)			Boys (n = 43 *)			Girls (n = 17 *)			Difference between Boys and Girls *p*-Value
	Not True % (n)	Somewhat True % (n)	Certainly True % (n)	Not True % (n)	Somewhat True % (n)	Certainly True % (n)	Not True % (n)	Somewhat True % (n)	Certainly True % (n)	
Considerate of other people’s feelings	8.3 (5)	58.3 (35)	33.3 (20)	7.0 (3)	62.8 (27)	30.2 (13)	11.8 (2)	47.1 (8)	41.2 (7)	0.720
Restless, overactive, cannot stay still for long	6.7 (4)	26.7 (16)	66.7 (40)	2.3 (1)	20.9 (9)	76.7 (33)	17.6 (3)	41.2 (7)	41.2 (7)	0.004 ^Boys^
Often complains of headaches, stomach aches, or sickness	71.7 (43)	16.7 (10)	11.7 (7)	72.1 (31)	14.0 (6)	14.0 (6)	70.6 (12)	23.5 (4)	5.9 (1)	0.741
Often has temper tantrums or hot tempers *	54.2 (32)	23.7 (14)	22.0 (13)	52.4 (22)	23.8 (10)	23.8 (10)	58.8 (10)	23.5 (4)	17.6 (3)	0.592
Rather solitary, tends to play alone	50.0 (30)	43.3 (26)	6.7 (4)	51.2 (22)	41.9 (18)	7.0 (3)	47.1 (8)	47.1 (8)	5.9 (1)	0.866
Generally obedient, usually does what adults request	10.0 (6)	53.3 (32)	36.7 (22)	14.0 (6)	58.1 (25)	27.9 (12)	0.0 (0)	41.2 (7)	58.8 (10)	0.014
Many worries, often seems worried	51.7 (31)	35.0 (21)	13.3 (8)	53.5 (23)	39.5 (17)	7.0 (3)	47.1 (8)	23.5 (4)	29.4 (5)	0.159
Constantly fidgeting or squirming	6.7 (4)	21.7 (13)	71.7 (43)	0.0 (0)	18.6 (8)	81.4 (35)	23.5 (4)	29.4 (5)	47.1 (8)	<0.001 ^Boys^
Easily distracted, concentration wanders	3.3 (2)	18.3 (11)	78.3 (47)	2.3 (1)	9.3 (4)	88.4 (38)	5.9 (1)	41.2 (7)	52.9 (9)	0.007
Nervous or clingy in new situations, easily loses confidence **	40.0 (24)	36.7 (22)	23.3 (14)	44.2 (19)	34.9 (15)	20.9 (9)	29.4 (5)	41.2 (7)	29.4 (5)	0.301
Gets on better with adults than with other children	58.3 (35)	36.7 (22)	5.0 (3)	55.8 (24)	39.5 (17)	4.7 (2)	64.7 (11)	29.4 (5)	5.9 (1)	0.653
Many fears, easily scared	78.3 (47)	18.3 (11)	3.3 (2)	74.4 (32)	20.9 (9)	4.7 (2)	88.2 (15)	11.8 (2)	0.0 (0)	0.205
Sees tasks through to the end, good attention span	58.3 (35)	30.0 (18)	11.7 (7)	74.4 (32)	23.3 (10)	2.3 (1)	17.6 (3)	47.1 (8)	35.3 (6)	<0.001 ^Girls^

Note: Bonferroni correction was applied, and the significance level was set to 0.004. ^Boys^ = Boys being assessed as having these behaviors more often than girls. ^Girls^ = Girls being assessed as having these behaviors more often than boys. * Indicates 10 missing scores, 8 boys and 2 girls. ** Total n = 59, boys n = 42, one missing.

**Table 3:** Teachers’ assessments of whether the challenges interfered with the children’s everyday life on the Strengths and Difficulties Questionnaire and differences between boys and girls (N = 70).

	Total (N = 58 *)				Boys (n = 42 *)				Girls (n = 16 *)				Difference between Boys and Girls *p*-Value
	Not at All % (n)	Only a Little % (n)	Quite a Lot % (n)	A Great Deal % (n)	Not at All % (n)	Only a Little % (n)	Quite a Lot % (n)	A Great Deal % (n)	Not at All % (n)	Only a Little % (n)	Quite a Lot % (n)	A Great Deal % (n)	
Distress the pupil	17.2 (10)	44.8 (26)	27.6 (16)	10.3 (6)	19.0 (8)	47.6 (20)	23.8 (10)	9.5 (4)	12.5 (2)	37.5 (6)	37.5 (6)	12.5 (2)	0.313
Affect friendships	10.3 (6)	37.9 (22)	34.5 (20)	17.2 (10)	9.5 (4)	42.9 (18)	31.0 (13)	16.7 (7)	12.5 (2)	25.0 (4)	43.8 (7)	18.8 (3)	0.596
Affect learning	3.4 (2)	34.5 (20)	44.8 (26)	17.2 (10)	0.0 (0)	28.6 (12)	50.0 (21)	21.4 (9)	12.5 (2)	50.0 (8)	31.3 (5)	6.3 (1)	0.007 ^Boys^
Affect classroom environment	12.1 (7)	31.0 (18)	39.7 (23)	17.2 (10)	4.8 (2)	31.0 (13)	45.2 (19)	19.0 (8)	31.3 (5)	31.3 (5)	25.0 (4)	12.5 (2)	0.026 ^Boys^

* Indicates missing scores (n = 12), 9 boys and 3 girls. ^Boys^ = Boys being assessed as having these behaviors more often than girls.

**Table 4:** Results of the Five to Fifteen (FTF) questionnaire and differences between girls and boys (pupils, N = 70).

	Total Mean (SD)	Boys Mean (SD)	Girls Mean (SD)	*p*-Value
From parental survey (n = 66):				
Attention	1.43 (0.34)	1.46 (0.34)	1.34 (0.35)	0.214
Attentions affecting everyday life	1.83 (0.74)	1.79 (0.75)	1.94 (0.72)	0.444
Hyperactivity–impulsivity	1.24 (0.43)	1.23 (0.45)	1.25 (0.36)	0. 833
Hyperactivity–impulsivity affecting everyday life	1.67 (0.77)	1.60 (0.79)	1.83 (0.70)	0.282
Hypoactivity	0.95 (0.46)	0.98 (0.48)	0.88 (0.42)	0.417
Hypoactivity affecting everyday life	1.02 (0.77)	1.02 (0.84)	1.00 (0.59)	0.922
Planning	1.25 (0.56)	1.32 (0.52)	1.06 (0.62)	0.087
Planning affecting everyday life	1.47 (0.86)	1.56 (0.84)	1.22 (0.87)	0.154
Body perception	0.68 (0.46)	0.70 (0.46)	0.63 (0.47)	0.626
Body perception affecting everyday life	1.00 (0.84)	0.96 (0.82)	1.11 (0.90)	0.511
From teacher survey (n = 60):				
Reading/writing/math	0.69 (0.57)	0.75 (0.57)	0.55 (0.59)	0.226
Reading/writing/math affecting everyday life	0.93 (0.92)	0.95 (0.88)	0.88 (1.03)	0.773
General learning	0.73 (0.40)	0.79 (0.39)	0.57 (0.39)	0.061
General learning affecting everyday life	0.95 (0.86)	1.02 (0.82)	0.75 (1.00)	0.287
Coping skills in learning	1.05 (0.50)	1.18 (0.46)	0.73 (0.49)	0.002 ^Boys^
Coping skills in learning affecting everyday life	1.48 (0.87)	1.58 (0.82)	1.24 (0.97)	0.167

Note: 0 = does not apply, 1 = applies sometimes or to some extent, 2 = definitely applies. Higher scores indicate more difficulties/challenges. Bonferroni correction was applied, and the significance level was set to 0.003. *p*-value for the difference between girls and boys was calculated using the Fisher–Freeman–Halton exact test. ^Boys^ = Boys being assessed as having more challenges than girls.

**Table 5:** Possible effects after a three-week use of the vest on the challenges reported on the Five to Fifteen questionnaire (pupils N = 70).

	Total Mean (SD)	Boys Mean (SD)	Girls Mean (SD)	Difference between Boys and Girls *p*-Value
Parental survey (n = 62)				
Attention	0.41 (0.64)	0.50 (0.67)	0.18 (0.53)	0.082
Hyperactivity–impulsivity	0.37 (0.55)	0.40 (5.91)	0.29 (0.47)	0.510
Hypoactivity	0.22 (0.52)	0.19 (0.54)	0.29 (0.47)	0.580
Planning	0.26 (0.56)	0.30 (0.61)	0.15 (0.38)	0.414
Body perception	0.39 (0.57)	0.34 (0.60)	0.50 (0.52)	0.426
Teacher survey (n = 49)				
Reading/writing/math	0.29 (0.55)	0.32 (0.58)	0.20 (0.44)	0.675
General learning	0.30 (0.54)	0.32 (0.57)	0.20 (0.44)	0.660
Coping skills in learning	0.31 (0.57)	0.29 (0.60)	0.38 (0.52)	0.699

Note: −2 = much worse, −1 = a little worse, 0 = the same, 1 = a little better, 2 = much better.

**Table 6:** Association between the extent of use of the vest and progress regarding challenges as assessed by the Five to Fifteen questionnaire (n = 70).

Independent Variable	Within-Group Change, Mean (SD)	B (CI)	*p*-Value
Reference category: Zero-type use of the vest (n = 27)	0.96 (1.48)		
One-type use of the vest (n = 21)	0.90 (1.26)	−0.06 (−1.39, 1.28)	0.931
Two-type use of the vest (n = 13)	2.77 (3.03)	1.81 (0.26, 3.36)	0.023
Three-type use of the vest (n = 9)	3.44 (4.33)	2.48 (0.71, 4.25)	0.007

Linear mixed model regression analysis was applied. *Note*: Types of use—90 min at the beginning of every school day (one type), 90 min at the beginning of every school day and additionally at school or at home (two types), and 90 min at the beginning of every school day and additionally at school and at home (three types). The dependent variable was the assessment of the possible effects: much worse = −2, a little worse = −1, the same = 0, a little better = 1, much better = 2.

## Data Availability

The data that support the results of this study are available from Open Patient data Explorative Network; restrictions apply to the availability of these data, which were used under license for the current study, and so are not publicly available.
